# Comparison of Bioaugmentation and Semipermeable Cover as Strategies for Micro-Pollutant Removal in Sewage Sludge Composting

**DOI:** 10.3390/toxics13080620

**Published:** 2025-07-25

**Authors:** Gabriela Angeles-de Paz, Miguel Ángel Díaz-Moreno, Ángeles Trujillo-Reyes, Cristina Postigo, Elisabet Aranda, Concepción Calvo, Tatiana Robledo-Mahón

**Affiliations:** 1 Microbiology and Environmental Technologies Group, Institute of Water Research, University of Granada, 18071 Granada, Spain; gangeles@ugr.es (G.A.-d.P.); miguelangeldiaz090@gmail.com (M.Á.D.-M.); angeles.trujillo@ugr.es (Á.T.-R.); earanda@ugr.es (E.A.); ccalvo@ugr.es (C.C.); 2 Department of Microbiology, Pharmacy Faculty, University of Granada, Campus de Cartuja s/n, 18071 Granada, Spain; 3 Technologies for Water Management and Treatment Research Group, Department of Civil Engineering, University of Granada, Avda. Severo Ochoa s/n, 18071 Granada, Spain; cristina.postigo@ugr.es

**Keywords:** bioaugmentation, composting, emerging pollutants, phytotoxicity, sewage sludge

## Abstract

Untreated sewage sludge (SS) and misused stabilization technologies have contributed to great contamination and the accumulation of various pollutants in agricultural soils. Regarding micro-pollutants’ degradation, scalable and effective technologies are still scarce. Although many attempts at composting adaptations have been discussed, only a few have been tested individually under outdoor conditions. To investigate different composting methods (bioaugmentation and semipermeable cover) for the removal of micro-pollutants frequently found in SS, we performed a set of on-site experiments. Windrows of SS and olive pruning were used as the compostable material and were subjected to (i) bioaugmentation with the fungus *Penicillium oxalicum*, (ii) covered composting, (iii) covered and bioaugmented composting, and (iv) a conventional composting pile, which was included as a control. The entire experiment lasted 99 days. Bioaugmentation without cover increased the phosphorus content, favored a reduction in heavy metal content, and was the only treatment that reduced carbamazepine at the end of the process. Moreover, the inoculation of *P. oxalicum* under semipermeable cover increased the richness, diversity, and dominance of specific microbial taxa and total bacterial abundance. The four mature composts obtained met the standards required to be classified in the B fertilizer category, showing that we reduced most of the micro-pollutants, and passed the germination test.

## 1. Introduction

Given the growing global population and water use, a greater amount of sewage sludge (SS) is produced worldwide. In the European Union, about 15 millions of tons of SS were produced in 2021 alone [1]. Thus, there is an urgent need to address its management while updating the requirements of the current policies. According to the European Green Deal of the European Commission and the Updated Bio-Economy Strategy with the EU Circular Economy Action Plans, this wastewater treatment byproduct needs to be reinserted to ensure the circularity of the economy [1]. This means that treatments must be focused on increasing the value of this waste as much as possible rather than eliminating this waste by incineration, for instance. Due to the high contents of valuable compounds within SS (including plant nutrients, organic matter, recoverable materials, and so on), SS transformation into agronomic amendments has become one of the most promising approaches from a circular economy perspective. However, SS is a complex granular matrix whose physicochemical properties increase the sorption of hydrophobic substances like emerging and priority contaminants, which mean SS is considered a concentrated waste for a diverse range of micro-pollutants, including “pollutants of emerging concern” such as pharmaceutical and personal care products, pesticides, and/or illicit drugs [2,3].

Sludge is subjected to different strategies to transform it into a more hygienic waste (anaerobic digestion, composting, lime treatment) [4,5]. Among them, composting achieves a product with added economic and environmental value, producing a sanitized, mature compost with a high content of stabilized organic matter and macronutrients that can be used as fertilizer or as a soil amendment. Moreover, it has been reported that composting may reduce the concentrations of some emerging pollutants, such as pharmaceutical active compounds (PhACs) [6].

The composting process is regulated in Europe by Regulation (EU) 2019/1009 of the European Parliament and the Council, which provides safety and environmental standards [7]. It is important that the composting process is carried out accordingly since immature compost and final products exceeding the maximum limits established may cause phytotoxicity in crops and can form a source of diseases when applied in agriculture [8]. Composting is an aerobic process for the stabilization of organic matter whose efficacy relies mainly on microbial activity and succession [9,10,11]. As the main means of the process, microbial-focused strategies are a useful approach to improving the quality of the final compost as well as other aspects outside of regulation. In this sense, bioaugmentation is an strategy used in the composting process that consists of the application of specific microorganisms like bacteria or fungi, which increase the mineralization process and the concentrations of micro- and macronutrients [12] and accelerate the degradation of the composted waste, achieving stability and maturity sooner [13]. Promising results of bioaugmentation’s application in composting have been reported in numerous studies [3,6,14,15,16,17,18,19], though there are fewer studies than those regarding the degradation of PhACs in composting. In our previous study [6], we performed bioaugmented composting, obtaining a degradation of 21% of the total pharmaceuticals detected in composting, but not many studies have focused on other emerging pollutants.

Many bioaugmentation studies have been conducted in the laboratory under controlled parameters due to plant-scale, unexpected, and outdoor challenges. Moreover, only a few studies have focused on implementing specific strategies that improve the composting process while monitoring the emerging and organic pollutants or the phytotoxic effect on the soil. In addition, the operational time is often considered a drawback for both small- and large-scale operations causing nutrient leaching, odors, and toxic gases’ release to the atmosphere. However, the incorporation of other technologies like semipermeable covers could contribute to decreasing the composting time at a real scale, making the process quicker and more economical [20].

To address these shortcomings, the present study evaluates the viability of SS composting with the combined application of bioaugmentation and a semipermeable cover system, as well as their individual performances in terms of pollutant degradation. The composting strategy was based on the inoculation of *Penicillium oxalicum* XD 3.1, which has previously been tested and found to be a good inoculant that decreases many emerging pollutants [6], and the use of a semipermeable cover system that has previously been tested and found to be an effective strategy in reducing the composting time [20]. Taking an intermediate approach between laboratory tests and real-scale implementation, composting was carried out at a pilot scale under real conditions in a biosolid plant.

## 2. Materials and Methods

### 2.1. Composting Strategies

*Inoculum preparation of Penicillium oxalicum*

The fungus *P. oxalicum* XD 3.1 belongs to the fungal collection of Dr. Elisabet Aranda at the University of Granada and it was isolated from a hydrocarbon-polluted pond [21]. The inoculum-obtaining process and concentration adjustments for SS composting were standardized and described by Angeles-de Paz et al. [6]. Briefly, the fungus was first massively cultivated on Petri dishes contained Malt Extract Agar medium (MEA, VWR chemicals, Radnor, PA, USA) and the spores were harvested, collected, and stored at 4 °C until their application. Spores’ concentrations were adjusted to 6.25 × 10^9^ spores kg^−1^ of sludge.

*The Semipermeable Cover Membrane*

The semipermeable cover film was an FR-COMPOSPLY cover purchased from INCABO, S.A. (Canovelles, Barcelona, Spain) (www.incabo.com). It is composed of three layers: two external Polyester HT Taslan fabric and a membrane PTFE between them. The respirability is set as >4000 g/m^2^/24 h, which indicates a moderate level of breathability. More details of the product are provided by the manufacturers.

### 2.2. Experimental Design

The composting piles were built at the facilities of the Environmental Complex EIDER recycling Eco-industry located in Guadix, Granada, Spain (latitude and longitude coordinates: 37.32583820223778, −3.08280105397221). The initial mixture consisted of approximately 3200 kg of digested SS (previously dehydrated by centrifugation in the wastewater treatment) and 9600 kg of olive pruning wastes, used as the bulking agent (B) (both received as wastes at the biosolid plant and regularly routinely treated at their facilities). After homogenization, the initial mixture was divided into four similar piles with the help of a shovel truck. The dimensions of each pile were around 2.5 m (L) × 1.5 m (W) × 1.3 m (H). Three out of the four piles were subjected to a different strategy while the remaining one worked as the composting control (conventional methodology). The treatments were labeled as follows: CP: Control Pile (traditional composting without cover nor inoculant), CoP: Covered Pile, PeP: *Penicillium* Pile (bioaugmented with *Penicillium oxalicum* XD 3.1), and CoPeP: Covered *Penicillium* Pile (bioaugmented with *Penicillium oxalicum* XD 3.1 and covered using the described semipermeable membrane). The four piles were set up simultaneously.

The composting experiments were conducted from February to May 2023. Inoculation was applied to both bioaugmented piles (PeP and CoPeP) at 0, 7, 21, and 30 days with the spore suspension dissolved in 10 L of tap water, while the other piles (CP and CoP) were watered with 10 L of tap water each. The latter were uncovered after 44 days of composting and left exposed until maturation (99 days). The four piles were regularly and mechanically turned over and monitored during the experiment. Sample collection was carried out throughout the experiment (at 0, 7, 14, 21, 30, 44, 66, and 99 days). Each composite sample comprised 800 g of compost obtained after mixing and homogenized small sub-samples from the four major zones (upper, outer, inner, and lower zones) within each pile (composite-type samples). Each composite was divided into five zip-loc bags, labeled according to the analyses performed, and stored at 4 °C or −20 °C, accordingly.

### 2.3. Physicochemical Analysis and Quantification of Viable Microorganisms

*Physicochemical Parameters*

The temperature of each pile was recorded with a temperature probe at the core of the piles. The storage composite at 4 °C, weighing 300 g, was analyzed by ACCIMESA S.L laboratories, to determine the humidity (H%), dry matter (DM%), total and volatile solids (ST—SV%), C/N ratio, and macronutrients from the solid fraction. The electrical conductivity (EC), pH, total organic matter (TOM%), and total carbon content (TCC%) were determined from the aqueous extract of the sample. All physicochemical parameters were determined following the Normalized Working Procedures described by *The Official Journal of the European Union* [22].

For the heavy metal determination, 100 g of composite was analyzed at the beginning of the composting (initial mixture) and in the mature compost (mature compost), and Cu, Zn, Pb, Cd, and Ni were processed with HNO_3_ + HCl using flame atomic absorption spectrometry (PerkinElmer, Waltham, MA, USA) as established in the Spanish version of the European CEN methods of UNE-EN 13650 [23]. Total mercury (Hg) was assessed with a direct analysis using thermal decomposition, amalgamation, and atomic absorption spectrometry by following Method EPA 7473 [24]. Hexavalent chromium (Cr VI) was measured spectrophotometrically at 540 nm using a UV-Vis Spectrophotometer (Agilent, Santa Clara, CA, USA) as previously described by Angeles-de Paz et al. [6]. Compost quality classification based on the heavy metal concentration was performed according to Spain’s Royal Decree 506/2013, which regulates the fertilization of products, including compost derived from organic waste. Additionally, heavy metal content results were used for removal/leaching rate calculation considering the original concentrations within the initial mixture.

*Quantification of Viable Microorganisms*

Tenfold serial dilutions were prepared from 1 g of each composite on the same day of the sampling. An aliquot from 10^−3^–10^−4^ dilutions in 0.45% saline solution was plated on Yeast Malt Extract Agar (YMC; 10 g L^−1^ malt extract, 2 g L^−1^ yeast extract, 20 g L^−1^ agar) supplemented with tetracycline (50 µg mL^−1^) and streptomycin (50 µg mL^−1^) for fungal growth, while 10^−6^ to 10^−7^ dilutions were plated on Trypticase Soy Agar (TSA, Oxoid©, Madrid, Spain) supplemented with cycloheximide (50 µg mL^−1^) for bacterial growth. YMC Petri dishes were incubated at 28 °C for 5–7 days, and TSA Petri dishes were incubated over 24–48 h at 30 °C. The colonies were counted and expressed as colony-forming units per gram of sample (CFU g^−1^)

### 2.4. Emerging Pollutants’ Determination

For emerging pollutants’ determination, the samples were extracted according to the methodology described by Montemurro et al. [25] with some modifications. Briefly, 0.5 g of freeze-dried and sieved samples (particle size < 500 μm) were spiked with internal standard compounds at 2 ng g^−1^ to analyze the yield of the extraction. Samples were hydrated with EDTA-McIlvaine buffer and further extracted with acetonitrile solvent. Subsequently, PSA and C18 adsorbent, followed by the QuEChERS methodology, were used for purification. After evaporation with nitrogen steam, the samples were reconstituted with 1.0 mL water/MeOH (90:10 *v*/*v*). Triplicate samples were analyzed using an Agilent 6470A, triple quadrupole mass spectrometer (LC-MS/MS) in the electrospray ionization positive ion mode (ESI+) via multiple reaction monitoring (MRM), available at the Institute of Water Research.

### 2.5. Quantitative Polymerase Chain Reaction (qPCR) Analysis

Samples intended for DNA extraction were cooled to −20 °C. Briefly, 500 mg of each sample was distributed in triplicate into screw-cap bead vials from the FastPrep^®^ Soil DNA Extraction Kit (MP Biomedicals, Santa Ana, CA, USA) and processed according to the manufacturer’s instructions. The DNA concentration was quantified using a NanoDrop 2000 spectrophotometer (Thermo Fisher Scientific, Waltham, MA, USA).

Bacterial gene expression levels in compost piles were assessed by qPCR using DNA extraction products in triplicate across three 96-well microplates, employing a bacterial-specific plasmid containing the 16S rRNA gene as a standard. The assays were carried out in DreamTaq HotStart Taq Master Mix (Thermo Fisher Scientific, Waltham, MA, USA) with a total volume of 25 μL. The qPCR reaction mixtures contained 0.125 μL of DNA polymerase (50 U μL^−1^), 0.15 μL of each primer (10 μM), 2.5 μL of Taq Buffer (10×), 0.5 μL of dNTPs (10 mM), 0.0625 μL of bovine serum albumin (BSA, 20 mg mL^−1^), and 0.125 μL 20× SYBR green diluted in DMSO and 2 μL of DNA template from ten-time-diluted samples of the original DNA. Primer sequences used for 16S ARNr amplification were P1-341F (5′-CCTACGGGAGGCAGCAG-3′) and P2-534R (5′-ATTACCGCGGCTGCTGG-3′) [26]. Calibration curves were constructed using linearized plasmid standards harboring inserts of the targeted sequences. Baseline values were set as the lowest fluorescence signal measured in the well over all cycles. The baseline was subtracted from all values, and the threshold was set to one standard deviation of the mean. All the qPCR runs included replicates and control reactions without a template. Based on the standard calibration curves, the total DNA content in the compost sample was expressed as the number of copies of 16S DNA g^−1^ of compost.

### 2.6. Amplicon Sequencing and Bioinformatic Analysis

About 25 µL of the obtained DNA was sequenced using Illumina MiSeq technology at the Institute of Parasitology “López Neyra” (Granada, Spain) using the following primer sets: ITS1 Fw (5′ GAACCWGCGGARGGATCA 3′) and Rv (5′ GCTGCGTTCTTCATCGATGC 3′). For bacteria, we used 16S ProV3V4 forward (5′ GCATCGATGAAGAACGCAGC 3′) and reverse (5′ TTACTTCCTCTAAATGACCAAG 3′). For the raw fungal sequences, primers were removed using the cutadapt algorithm. Both raw bacterial sequences and fungal trimmed sequences were quality-filtered, dereplicated, chimera-screened, and merged for the amplicon sequence variants (ASVs), obtained using DADA2 ITS pipeline v.1.8 and DADA2 pipeline v1.8 for fungi and bacteria, respectively [27]. Taxonomic assignment of the ASVs was conducted using the UNITE ITS v8.3 database and the database SILVA v138.1 for 16S ARN.

### 2.7. Germination Index Tests

Germination tests were performed using *Lepidium sativum* seeds. Extracts from each composite (storage at −20 °C) were prepared in Erlenmeyer flasks via the addition of distilled water at 1:1, 1:2, 1:5, and 1:10 *w*/*v*. The flasks were shaken at 250 rpm for 1 h at room temperature. Seed preparation was conducted by hydrating with tap water to imbibe the seeds (non-viable seeds were discarded). Twenty viable seeds were distributed on rounded filter paper fixed on Petri glass dishes (9 cm) impregnated with the composite extracted. Petri dishes were incubated for 48 h at 28 °C. A control with distilled water was also included in the test. The germinability index (GI%) [28] was calculated according to the following formula:

%GI = (RSG%) (RRG%)/100(1)

%RSG = G/G_0_ (100) (2)

%RRG = L/L_0_ (100) (3)

where %GI Equation (1) is calculated considering the relative seed germination (%RSG, Equation (2)) and the relative radicle growth (%RRG, Equation (3)). Both germinated seeds with the sludge extract (G) and the number of germinated seeds in the control dish (G_0_) were counted for the %RSG, while the radicle in the seeds germinated and the sludge extract (L) and the length of the radicle in the seeds germinated in the control dish (L_0_) were measured for the %RGG.

### 2.8. Statistical Analysis

All experiments consisted of triplicates, and their design was completely randomized. The presented data are shown as means ± standard deviations. SigmaPlot v.15.0 (Systat Software Inc., San Jose, CA, USA) was used for the statistical analysis. Significant differences (*p* < 0.005) were calculated via variance analyses (two-way ANOVA and one-way ANOVA) together with Duncan’s multiple range test, assessing normality and homoscedasticity, and considering replicates and repeated measurements. Additionally, the Kruskal–Wallis test by ranks was used for qPCR results’ evaluation.

## 3. Results and Discussion

### 3.1. Physicochemical Composition

The physicochemical and quality parameters are presented in Table 1. Parameters like pH were studied to determine the neutrality in all the piles at the beginning and at the end of the composting process. The EC increased for the four piles, reaching values higher than 1 dS m^−1^, following the trend of similar studies with SS under real conditions and using bioaugmentation strategies [19]. The content of moisture (%) decreased until reaching percentages under 10% at the end of the process for all the piles except for CoPeP, where it was 15.53 ± 0.96. In all the cases, the values required for the Spanish legislation were reached as well as the ratio of C/N. Regarding the content of heavy metals (Table 1), all the composts were classified as class B (according to Royal Decree 506/2013), indicating their potential for use in agronomy. P and K are, along with N, two of the most important macronutrients for plant development. The percentages of P_2_O_5_ and K_2_O_5_ increased in the mature compost; these values were higher in the piles with *Penicillum* treatment (PeP and CoPeP) while the percentage of P_2_O_5_ was higher in the CP and PeP piles than in the CP and CoPeP piles. The literature contains reports that the common values of P and K obtained in the final compost range between 2.2 and 3 g of K kg^−1^ of ST and between 3.2 and 3.8 g of P kg^−1^ of ST [29,30,31]. The concentrations of P and K obtained in this study were lower than in the literature, which can be explained by the low concentrations of P and K in the starting material.

The nutritional values of compost in PeP and CoPeP revealed the highest values for calcium (%CaO and %MgO). Regarding the %NTP content, the decrease in nitrogen in the treatments with bioaugmentation (PeP and CoPeP) could be related to the increase in enzymatic activity compared to the control and the higher increase in organic matter obtained. In the present study, *P. oxalicum* inoculation and semipermeable cover induced a higher macronutrient content than in bioreactor-enriched composting processes and traditional composting and commercial composting processes [32].

A general comparison among heavy metal reductions is shown in Table 2. In all treatments, a decrease in the concentration of metals was observed, except for Cu in the CoPeP pile, in which a slight increase was observed at the end of the experiment. The metals with the highest initial and final concentrations in all treatments were Zn and Cu, and the metals with the lowest initial and final concentrations were Hg and Cd.

Despite the mixture having been previously homogenized before it was divided into four piles, the initial concentration of heavy metals was different in each treatment. Hence, the percentage of reduction for heavy metals was calculated to compare the effect, whether of bioaugmentation or cover strategies. Cd, Hg, Pb, Cr, Cu, and Zn were more considerably reduced in the bioaugmented piles, independent of the use of the cover. In most of the cases, the limiting heavy metal was Cu; thus, to obtain A classification quality compost according to the Spanish regulation, complementary treatment would be needed. For instance, treatment with illite, a clay mineral, decreases the bioavailability of Cu during composting [33]. Another potential strategy to reduce the concentration of heavy metals could be the addition of more bulking agent. However, this approach might be disadvantageous, since it reduces the volume of waste (SS) being composted. Additionally, the initial content of pollutants can vary depending on the initial characteristics of the SS. In this sense, according to the final use of compost, different strategies may be more appropriate. It is important to point out that copper is commonly applied to soil by farmers in the form of inorganic salt, such as copper sulfate (CuSO_4_). Hence, these practices could potentially be replaced by compost with a naturally high copper content, as it has been shown that Cu from SS is more soluble than from inorganic salts, likely due to the presence of organic matter [34]. In summary, to decrease the percentage of heavy metals, treatment with *Penicillium* will be more successful to achieve this aim compared to the unique use of semipermeable cover or a combination, probably due to the adsorption capability of fungal mycelium.

### 3.2. Culturable Bacteria and Fungi

**Figure 1:**
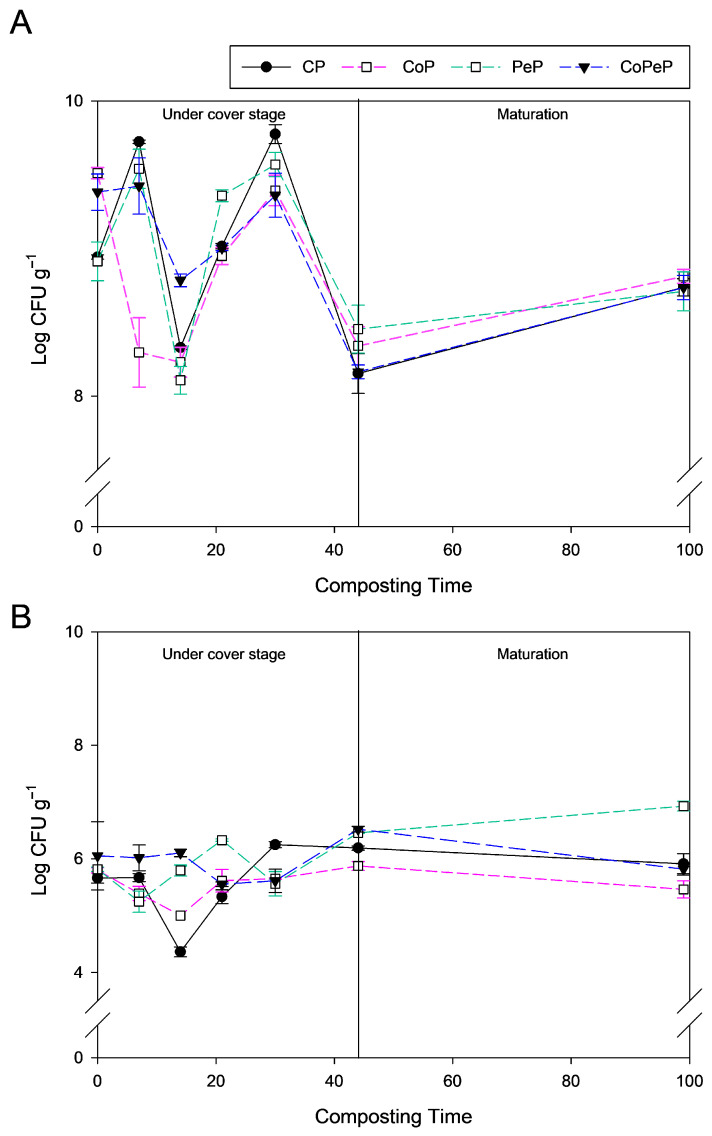
Culturable microorganisms: (**A**) bacterial log CFU g^−1^ and (**B**) fungal log CFU g^−1^ in the composite samples—CP (Control Pile), CoP (Cover Pile), PeP (*Penicillium* Pile), and CoPeP (Covered *Penicillium* Pile)—during the composting experiment. Error bars indicate the standard error of the mean (n = 3).

Figure 1 shows the CFU g^−1^ in all the piles. As has been mentioned, two phases can be distinguished according to the use of cover semipermeable film: a covered phase (from 0 to 44 days), which affected CoP and CoPeP, and a compost maturation phase (from 44 to 99 days). Bacteria dominated the culturable microbial community throughout the process, with concentrations ranging from 10^8^ to 10^10^ CFU g^−1^ of compost (Figure 1A). The fungal count was lower than the culturable bacteria, with concentrations between 10^4^ and 10^7^ CFU g^−1^ of compost (Figure 1B). Bacteria counting followed a decreasing trend during the covered phase and an increasing trend in the maturation phase until the end of the process in all treatments. Fungi in treatments CP, CoPeP, and CoP followed a positive trend in the covered phase and a negative trend in the maturation phase, while treatment with PeP maintained an increasing trend during both phases. In the pile CoPeP, a decline in bacterial and fungal populations was observed, possibly caused by the rapid temperature increase from 7 to 14 days (Figure S1). After 14 days, a rapid decrease in temperature was observed, coinciding with an increase in bacterial and fungal UFC until 30 days. From this point on, both microbial populations decreased until the cover was removed. In the maturation phase in pile CoPeP, the culturable bacterial number increased while fungi decreased.

*Penicillium* piles (PeP and CoPeP) maintained the thermophilic phase for slightly longer than the control piles, which was consistent with an increase in the culturable fungal concentration during the thermophilic phase and during the compost maturation phase. The results obtained in this experiment show an increase in fungal concentration in all treatments when the pile temperature decreased to 35 °C. The study by Robledo-Mahón et al. [35] obtained bacterial concentrations of 10^14^–10^18^ CFU g^−1^ of compost and 10^3^–10^14^ CFU g^−1^ of compost for fungi in a covered pile built to the real scale, which reached 80 °C and whose covered phase lasted 30 days. González et al. [36] showed in their results bacterial and fungal measurements of 10^8^ CFU g^−1^ of compost in a covered pile with temperatures slightly lower than the study by Robledo-Mahón et al. [35] but with a more extensive covered phase of 60 days. The results of this experiment showed bacterial concentrations in a similar range to that described by González et al. [36].

Bioaugmentation and cover treatment affected the culturable fungi counting in the inoculation step; moreover, the culturable bacterial count was increased in the uncovered pile in contrast to the covered ones. The followings steps showed an attenuation or even a boost in fungal count in bioaugmentation piles (PeP and CoPeP), in contrast to counting bacteria, whose evolution was similar in subsequent stages. Microbial dynamics were glossed over for the cover piles, with the highest stable physicochemical parameters in this treatment preventing sudden changes in the population. The stable fungal community was unchanged by the inoculation of external fungi; in contrast, *P. oxalicum* inoculation boosted the fungal population during the thermophilic step.

### 3.3. Micro-Pollutant Concentration

**Figure 2:**
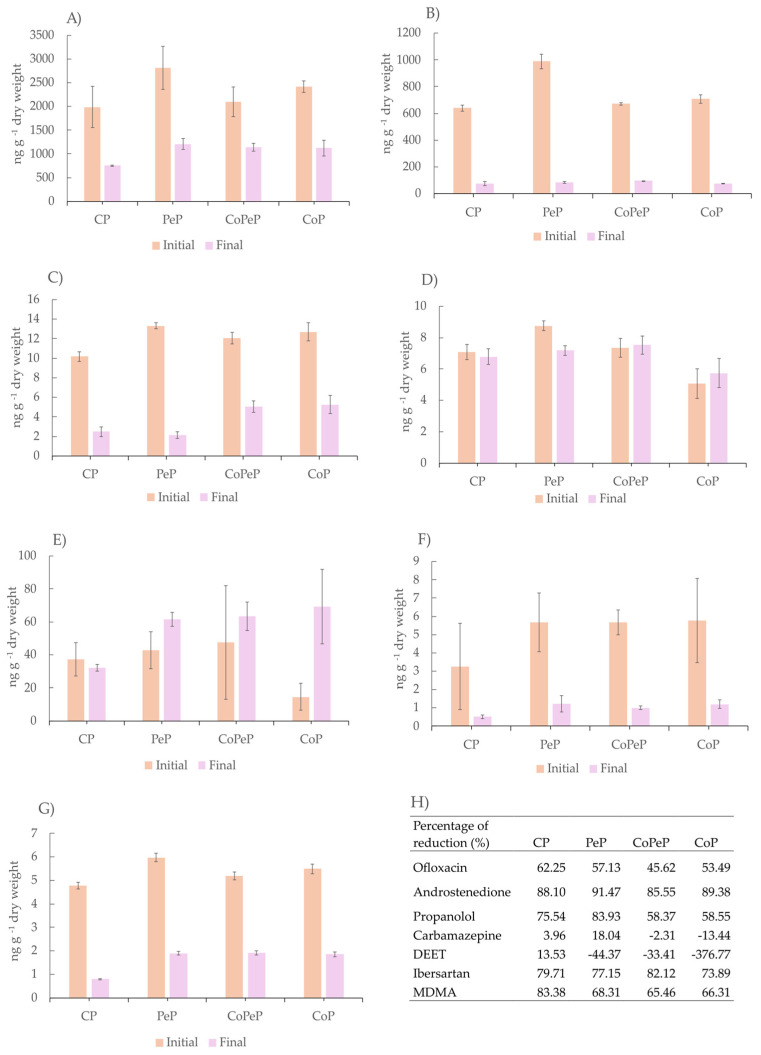
Target micro-pollutants: (**A**) ofloxacin, (**B**) androstenedione, (**C**) propranolol, (**D**) carbamazepine, (**E**) DEET, (**F**) irbesartan, and (**G**) MDMA at the beginning and at the end of the composting time contained in the four piles: CP (Control Pile), CoP (Cover Pile), PeP (*Penicillium* Pile), and CoPeP (Covered *Penicillium* Pile). (**H**) Summary table of the percentage of reduction for each determined compound.

The method described in Section 2.4 was used to determine a target group of seven micro-pollutant representatives of PhACs, like antibiotics (ofloxacin), hormones (androstenedione), beta-blockers (propranolol), blood pressure drugs (irbesartan), anti-epileptics (carbamazepine), and other micro-pollutants like insect repellent (DEET) and illicit drugs (MDMA). The selection of these diverse compounds allowed us to see the occurrence in the initial mixture and to monitor how models of micro-pollutants can be affected by composting (Figure 2). The compound with a higher concentration was the antibiotic ofloxacin (Figure 2A), which was found in a concentration higher that 200 ng g^−1^ in all the starting mixtures for the four composting piles. The hormone androstenedione (Figure 2B) was found in the range of 600–900 ng g^−1^ in the four piles and obtained the highest reduction in PeP (91.45%). The pesticide DEET was found in a concentration lower than 100 ng g^−1^ (Figure 2E), as was the case for propanol (Figure 2C), while carbamazepine (Figure 2D), irbesartan (Figure 2F), and MDMA (Figure 2G) were found at under 10 ng g^−1^ [6].

All the target monitored micro-pollutants decreased in mature compost in the four piles except for carbamazepine and DEET. Carbamazepine was reduced just in PeP, with bioaugmentation and no cover. A previous study also reported a reduction in carbamazepine by *P. oxalicum* in composting, also without cover. It has previously been reported that carbamazepine can be degraded by *P. oxalicum* through absorption into biomass [37]. However, other studies suggest that this degradation may also occur via enzymatic mechanisms, specifically by CYP450, which has been identified in this fungus [21]. Likely, the degradation of this compound in PeP involves a combination of factors, including absorption into biomass, CYP450 activity, and cooperation with other native microbes in compost. This is supported by evidence showing that *P. oxalicum* XD 3.1 can degrade this compound in consortium but not effectively on its own [38]. Understanding the reduction of recalcitrant compounds such as carbamazepine at a real scale remains a significant challenge, and more studies are needed to elucidate the combinatorial mechanisms involved in this process. Regarding DEET, researchers have studied its biodegradation by some fungi with limited capacities for its biotransformation [39]. However, *P. oxalicum* has not previously been reported for the degradation of this compound.

### 3.4. Microbial Succession During Composting

**Figure 3:**
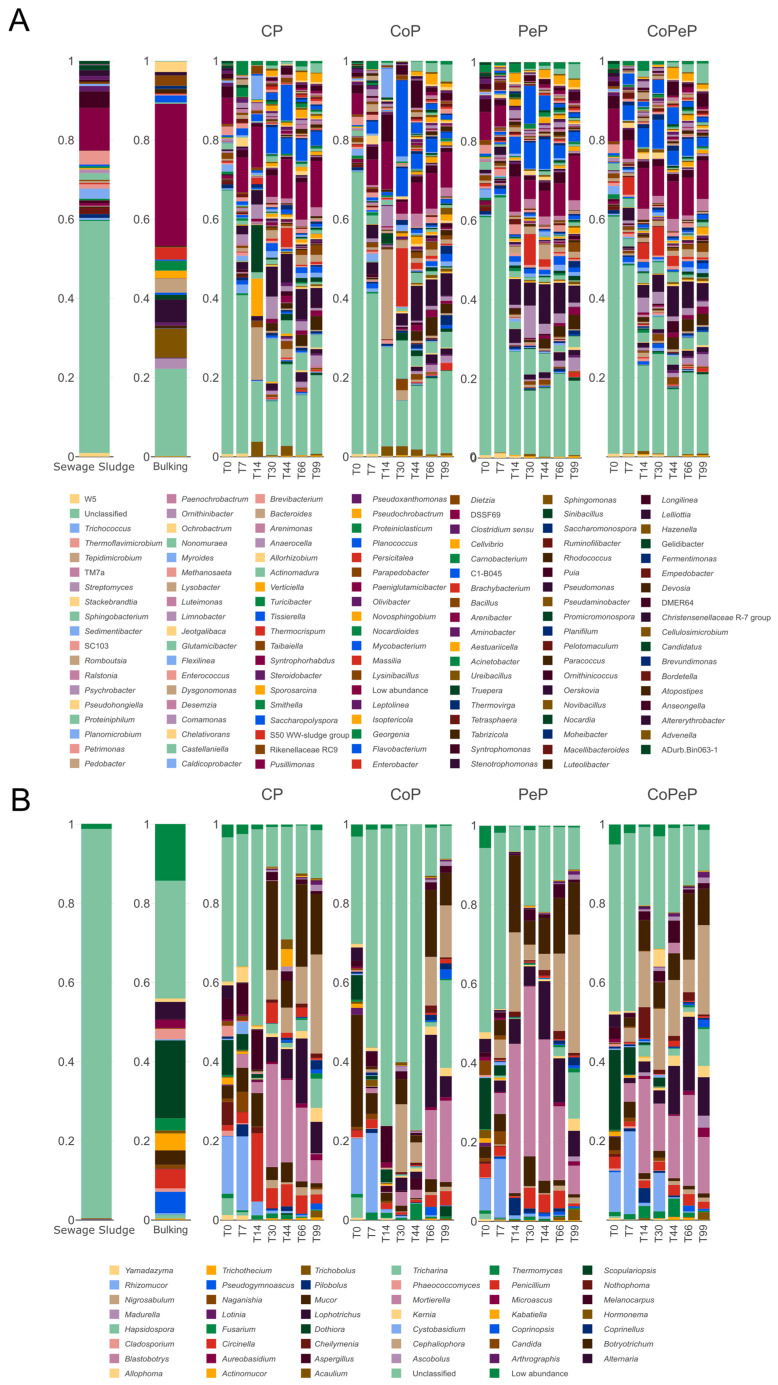
Relative abundance (%) of bacterial (**A**) and fungal (**B**) succession in all composites throughout the composting experiment: CP (Control Pile), CoP (Covered Pile), PeP (Covered *Penicillium* Pile), and CoPeP (Covered *Penicillium* Pile).

Figure 3 presents the relative abundance for bacterial and fungal communities. The structure of the bacterial community (Figure 3A) shows a different profile according to the treatment of the pile. All the treatments started the process with a high percentage of unclassified community from both starting materials (sewage sludge and bulking), which did not start to be modulated until 14 days into the process (T14), depending on the pile. For instance, in the CP, *Verticiellea* and *Turicibacter* had more abundance at this point. In the CoP, *Tepidimicrobioum* was a genus shared with CP but in this case more abundant. The profiles of the bacterial community for PeP and CoPeP were quite similar, which may indicate that bioaugmentation did not affect the autochthonous bacterial communities and had more effect than the use of cover in this case.

The fungal community (Figure 3B) showed a different trend. Regarding the starting material, it is notable how most of the community of the SS is unclassified. This could mean that many of the fungal representatives of this type of waste have not yet been described and are, therefore, unknown in the field of fungal diversity. This also occurred in a lower proportion for the bulking agent, which was dominated by genera like *Aspergillus*, *Fusarium*, *Cladosporium Trichothecium*, *Pseudogymnoascus*, and *Pilobolus*, though a considerable proportion of unclassified members were still found. This part of the fungal community stayed throughout the process in all the piles, although the profile changed when comparing the time and the treatment. In this way, the use of cover (CP) decreased in some populations like *Blastrobotrys*; however, this was not affected in CoPeP when *Penicillium* was inoculated. The bioaugmentation with *Penicillium* was not clearly reflected in the relative abundance. Despite the surveillance of *Penicillium*, which colony was optically identified in the Petri dishes from microbial enumeration, the percentages of *Penicillium* in the different treatments were similar, and the genus *Penicillium* showed the highest percentage in the CP at 14 days (T14) compared to PeP or CoPeP. The fungal profile in mature compost (T99) showed a similar structure in all the treatments, showing more diversity and similar communities like *Acaulium*, *Allophoma*, *Blastrobotrys*, *Aureobasidium*, *Aspergillus*, and *Cephaliophora*.

### 3.5. Alpha Diversity Metrics During Composting

**Figure 4:**
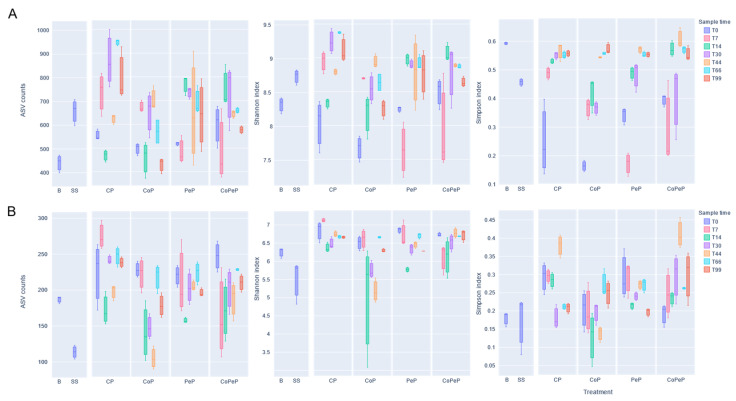
Bacterial (**A**) and fungal (**B**) ASV counts and diversity indexes from all composites throughout the composting experiment: CP (Control Uncover Pile), CoP (Cover Pile), PeP (*Penicillium* Pile), and CoPeP (Covered *Penicillium* Pile).

Peaks of richness (unique ASVs per sample), diversity (Shannon index), and dominance (Simpson index) were observed throughout the composting during the experiments (Figure 4). For instance, higher richness and diversity were observed under conventional conditions (CP), once the end of the thermophilic stage was observed (after T30, S1). As expected, fewer unique ASVs were found when the temperature reached its maximum (between T7 and T14). Dominancy between specific species followed and was shaped by the temperature fluctuations since each stage was characterized by different degradation activity, which forms the basis of composting technologies [40]. Compared to traditional composting, the use of semipermeable cover is expected to reduce drawbacks associated with odors, emissions, and other physicochemical parameters. Although its use has been demonstrated to increase the richness of thermophilic species [20], the diversity, abundance, and evenness of microbial communities normally abate because of extreme temperatures [41]. However, only microbial richness was negatively affected by the semi-permeable cover used for this study (regardless of the longer thermophilic period, which lasted until T44). The diversity results were indeed similar to those observed in CP.

While diversity and richness fluctuated during the CP and CoP treatments, both bioaugmented piles showed a more stable trend in microbial populations, regardless of the physicochemical conditions, including temperature, which in particular depleted many fungal species. The presence of *P. oxalicum* showed signs of resilience indirectly contributing to other species by improving physicochemical conditions like EC [42] or N availability [42,43], achieved by decreasing diverse contaminants’ concentrations, such as heavy metals and PhACs [6,44,45]. These results prove the stability, consistency, and performance in terms of populations dynamics of this bioaugmentation methodology [6]. They also confirm the adaptability of its inoculation regardless of the composting time, scalability, and pollutants initial occurring. With PeP and CoPeP, covered technology seemed to improve the dominancy of certain bacterial (during the thermophilic stage) and fungal species (at the beginning of the maturation stage), which might indicate earlier signs of maturation.

### 3.6. Bacterial Abundance (qPCR)

The quantitative polymerase chain reaction (qPCR) monitors the increase in fluorescence per cycle during the amplification of DNA in real-time; this progressive rise in fluorescence enables the determination of the gene copy number, which relies on the linear relationship between the logarithm of initial template quantity and the quantification cycle (Cq) value observed in the amplification [46].

**Figure 5:**
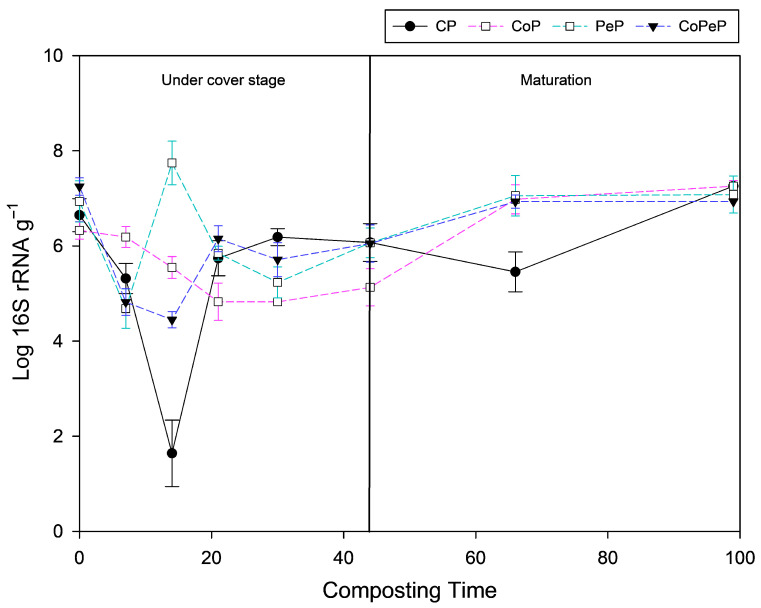
Number of copies of 16S rRNA from genomic DNA of composting samples for CP (Control Uncover Pile), CoP (Cover Pile), PeP (*Penicillium* Pile), and CoPeP (Covered *Penicillium* Pile).

The determination of the gene copy number for the piles is shown in Figure 5. The gene abundance shows a range of 10^5^ to 10^13^ gene copies with a good linearity and accurate R^2^ (0.9919). The gene copies descended in all the piles during the thermophilic phase, except for the PeP piles, which increases the bacteria in the compost at 14 days. The gene copies increased in PeP on day 14. Regarding the culturable bacteria (CFU g^−1^ of compost), the low count at this stage may indicate that most of the microbial population was unculturable bacteria or that the condition used (30 °C) was not optimal for their growth. Although this data may appear inconsistent, a decrease in culturable bacteria is not indicative of a reduction in microbial activity. Previous studies have shown that the highest microbial activity, including enzymatic activity, occurs during the thermophilic stage of composting [35,47,48]. Therefore, the use of complementary molecular techniques to quantify microbial communities provides a wider perspective on the composting process. However, further studies are needed to better understand the functional and active microbial groups involved in composting. The efficiency of the composting is often directly associated with temperature; consequently, the quality of compost could be improved by prolonging the thermophilic phase and the microbial activity of thermophilic microorganisms [47]. The active gene copies were more stable in PeP, which did not undergo significant variations during the maturation phase, unlike CoP which decreased in its copy number after the thermophilic phase and ended up with the highest copy number at the end of the maturation phase of all the piles. The gene copies of 16S had a significant reduction in CoPeP in the mature compost, which differed from the abundance of culturable bacteria in the pile at this time. In the study by Sosa et al. [49], as well as in our results, a decrease in the number of active DNA copies was observed in compost containing olive mill waste (alperujo). This may be since, in very mature compost, most bacteria are likely viable but in a latent state or with low metabolic activity. Therefore, they grow well on rich media such as TSA, contrary to their low amplifiable DNA or rRNA, reflecting a state of reduced replication or transcription [50]. This may also indicate that the compost is in a very stable phase, with generally low microbial metabolic activity [51].

### 3.7. Phytotoxicity Tests

**Figure 6:**
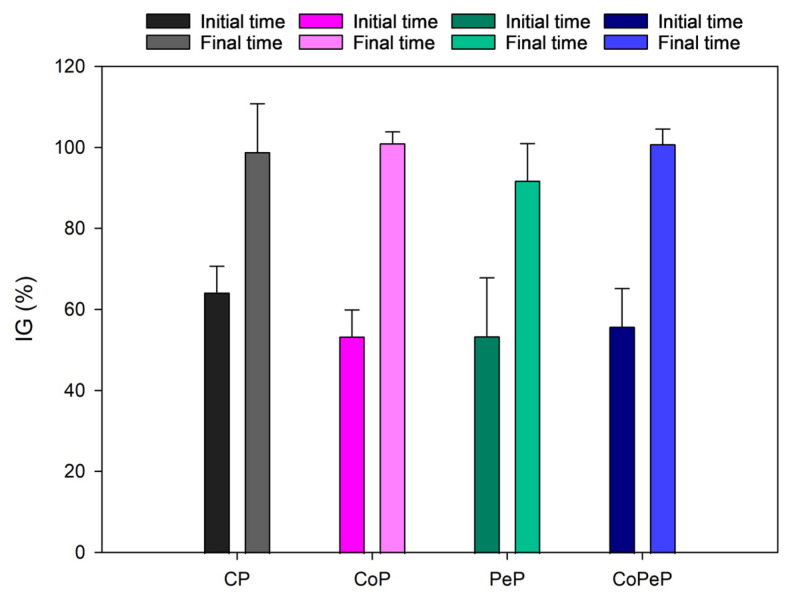
Phytotoxicity of composite samples from all treatments at initial and final composting times for CP (Control Uncover Pile), CoP (Cover Pile), PeP (*Penicillium* Pile), and CoPeP (Covered *Penicillium* Pile). Error bars indicate standard errors of the mean (*n* = 3).

The seed germination test was evaluated from each pile at the beginning and at the end of the process (Figure 6). A phytotoxicity test is an effective bioassay to evaluate the potential toxicity of compost before it is applied to the soil for plant growth [52]. The results showed that all the piles increased in %IG at the end of the composting process, reaching values higher than 90%. In the case of CoP and CoPeP, these values were higher than 100%.

The high values of %IG with the physicochemical parameters can be used to establish that all the piles reached maturity at the end of the process. Maturity refers to the degree of completion of the composting process [53]. The presence of compounds like ammonia and other phytotoxic compounds in high quantities would affect seed germination. Maturity is not only defined by phytotoxicity tests, but this can be considered one more parameter that confirms the efficacy of the process and the possibilities for using compost for agricultural purposes.

## 4. Conclusions

Bioaugmentation and the use of cover are well-known for obtaining a high-quality final compost. In this study, we assessed physicochemical and microbiological parameters, heavy metals, and micro-pollutants under four different strategies of composting (using both bioaugmentation and semipermeable cover, independently and synergistically). The comparative analysis between the initial material and the mature compost under all treatments revealed that the inoculation with *P. oxalicum* eased many physicochemical and biological factors, regardless of the use of semipermeable cover. For instance, although the use of the latter contributed to better moisture retention, inoculation increased the contents of specific macronutrients like phosphorus, diversity metrics of both bacteria and fungi, as well as bacterial total abundance of active populations. In spite of these differences, all four mature composts met the required standards to be classified in the B category of fertilizer. Furthermore, germination test results and emerging pollutants showed better values under all strategies tested.

## Figures and Tables

**Table 1:** Physicochemical and quality parameters of composite samples from each pile (CP: Control Pile, CoP: Covered Pile, PeP: *Penicillium* Pile, and CoPeP: Covered *Penicillium* Pile) at the beginning of the composting within the starting material (SM) and at the end of the process within the mature compost (MC). ± indicates standard deviation of 3 replicates (n = 3).

		CP		CoP		PeP		CoPeP	
		SM	MC	SM	MC	SM	MC	SM	MC
General parameters	pH	7.57	7.37	7.33	7.30	7.57	7.10	7.50	7.20
	EC (dS m^−1^)	0.56 ± 0.03	1.18 ± 0.01	0.56 ± 0.02	1.06 ± 0.01	0.67 ± 0.04	1.16 ± 0.03	0.61± 0.02	1.10 ± 0.03
	M (%)	56.20 ± 1.11	5.53 ± 0.34	55.00 ± 1.45	8.83 ± 0.79	60.63 ± 3.81	6.13 ± 0.38	59.76 ± 1.47	15.53 ± 0.96
	DM (%)	43.80 ± 1.11	94.5 ± 0.35	45.00 ± 1.45	91.16 ± 0.79	39.36 ± 3.81	93.86 ± 0.38	40.23 ± 1.47	84.46 ± 0.96
	TS (%)	44.00 ± 1.00	94.66 ± 0.33	45.33 ± 1.33	91.33 ± 0.88	39.33 ± 3.93	93.66 ± 0.33	40.33 ± 1.45	84.66 ± 0.88
	VS (%)	25.33 ± 0.88	42.33 ± 0.67	27.00 ± 1.00	33.33 ± 0.33	22.66 ± 2.33	40.66 ± 0.33	23.66 ± 0.88	34.00 ± 0.58
Macronutrients (%)	NTK (%)	2.25 ± 0.04	2.34 ± 0.08	2.42 ± 0.05	2.01 ± 0.08	2.86 ± 0.01	2.35 ± 0.07	2.55 ± 0.04	2.18 ± 0.03
	P_2_O_5_ (%)	2.26 ± 0.03	1.82 ± 0.04	2.40 ± 0.05	1.94 ± 0.06	3.05 ± 0.05	2.50 ± 0.12	2.58 ± 0.03	2.64 ± 0.10
	K_2_O_5_ (%)	0.50 ± 0.01	0.40 ± 0.02	0.50 ± 0.02	0.48 ± 0.02	0.47 ± 0.01	0.46 ± 0.01	0.51 ± 0.01	0.45 ± 0.01
	CaO (%)	9.30 ± 0.35	13.45 ± 0.88	9.55 ± 0.55	14.47 ± 0.82	8.77 ± 0.42	13.76 0.20	10.20 ± 0.41	12.21 ± 0.46
	MgO (%)	1.44 ± 0.03	1.66 ± 0.08	1.70 ± 0.05	1.91 ± 0.03	1.55 ± 0.06	1.62 ± 0.04	1.67 ± 0.03	1.73 ± 0.03
Organic compounds	TOM (%)	25.60 ± 0.83	34.00 ± 0.78	26.86 ± 1.02	29.41 ± 0.33	22.80 ± 2.40	31.30 ± 0.42	23.63 ± 0.95	28.71 ± 0.52
	DOM (%)	58.50 ± 1.50	50.24 ± 0.69	59.66 ± 0.33	50.63 ± 0.61	57.80 ± 0.70	51.02 ± 0.57	58.73 ± 0.66	50.05 ± 0.67
	TOC (%)	14.83 ± 0.50	19.72 ± 0.47	15.60 ± 0.60	17.05 ± 0.20	13.23 ± 1.39	18.15 ± 0.23	13.73 ± 0.54	16.65 ± 0.29
	DOC (%)	33.90 ± 0.90	29.12 ± 0.40	34.60 ± 0.21	29.34 ± 0.38	33.53 ± 0.42	29.58 ± 0.31	34.06 ± 0.38	29.02 ± 0.37
	MM (%)	18.20 ± 0.90	33.81 ± 0.46	18.13 ± 0.44	28.80 ± 1.04	16.60 ± 1.40	30.05 ± 0.70	16.56 ± 0.62	28.50 ± 1.00
	C/N	15.03 ± 0.32	13.54 ± 0.32	14.20 ± 0.51	12.06 ± 0.46	11.76 ± 1.29	12.18 ± 0.28	13.30 ± 0.03	12.93 ± 0.03
Heavy metals (mg kg^−1^)	Zn	525.33 ± 39.72	258.67 ± 8.65	421.33 ± 51.81	186.33 ± 6.33	822.33 ± 166.5	234.33 ± 6.12	669.00 ± 186.71	212.00 ± 49.52
	Cu	276.00 ± 26.72	141.67 ± 4.41	208.33 ± 22.26	216.00 ± 19.80	372.67 ± 93.49	138.00 ± 4.93	283.00 ± 64.28	138.00± 5.56
	Cr	75.00 ± 12.42	52.00 ± 4.04	51.33 ± 7.33	14.33 ± 0.88	90.00 ± 10.26	14.67 ± 0.88	73.00 ± 24.45	16.00 ± 14.31
	Ni	51.33 ± 11.02	39.00 ± 2.12	38.33 ± 9.29	14.33 ± 1.15	67.67 ± 18.23	33.00 ± 1.41	33.00 ± 1.41	63.00 ± 25.45
	Pb	44.00 ± 2.00	26.00 ± 0.03	38.00 ± 0.44	22.00 ± 1.04	74.67 ± 19.55	24.67 ± 0.67	55.00 ± 2.08	24.67 ± 0.88
	Hg	0.53 ± 0.03	0.30 ± 0.06	0.47 ± 5.69	0.20 ± 0.03	0.77 ± 0.15	0.27 ± 0.03	0.67 ± 0.03	0.27 ± 0.03
	Cd	0.67 ± 0.03	0.50 ± 0.03	0.56 ± 0.07	0.50 ± 0.03	0.73 ± 0.43	0.50 ± 0.03	0.80 ± 0.03	0.50 ± 0.03
Classification RD 506/2013			B		B		B		B

EC: electrical conductivity, M: moisture, DM: dry matter, TS: total solids, VS: volatile solids, TOM: total organic matter, DOM: dry organic matter, TOC: total organic carbon, DOC: dry organic carbon, MM: mineral matter.

**Table 2:** Heavy metal reduction (%).

		CP	CoP	PeP	CoPeP
Heavy metal	Zn	50.76	71.50	68.31	55.78
reduction	Cu	48.67	62.97	51.24	46.24
%	Cr	30.67	83.70	78.08	72.08
	Ni	24.03	51.23	29.41	62.61
	Pb	40.91	66.96	55.15	42.11
	Hg	43.75	65.21	60.00	57.15
	Cd	25.00	31.82	37.50	11.77

## Data Availability

Data is contained within the article or Supplementary Materials.

## Supplementary Materials

The following supporting information can be downloaded at https://www.mdpi.com/article/10.3390/toxics13080620/s1, Figure S1: Temperature over composting time under the four treatments: CP: Control Pile, CoP: Covered Pile, PeP: Penicillium Pile, and CoPeP: Covered Penicillium Pile.
