# Supplementary material for: Comparison of Bioaugmentation and Semipermeable Cover as Strategies for Micro-Pollutant Removal in Sewage Sludge Composting
Source: Toxics. 2025 Jul 25;13(8):620. doi: 10.3390/toxics13080620 (PMC12390589; doi:10.3390/toxics13080620)
Supplement: Supplementary file 1 [file toxics-13-00620-s001.zip › toxics-3692248-supplementary.pdf]

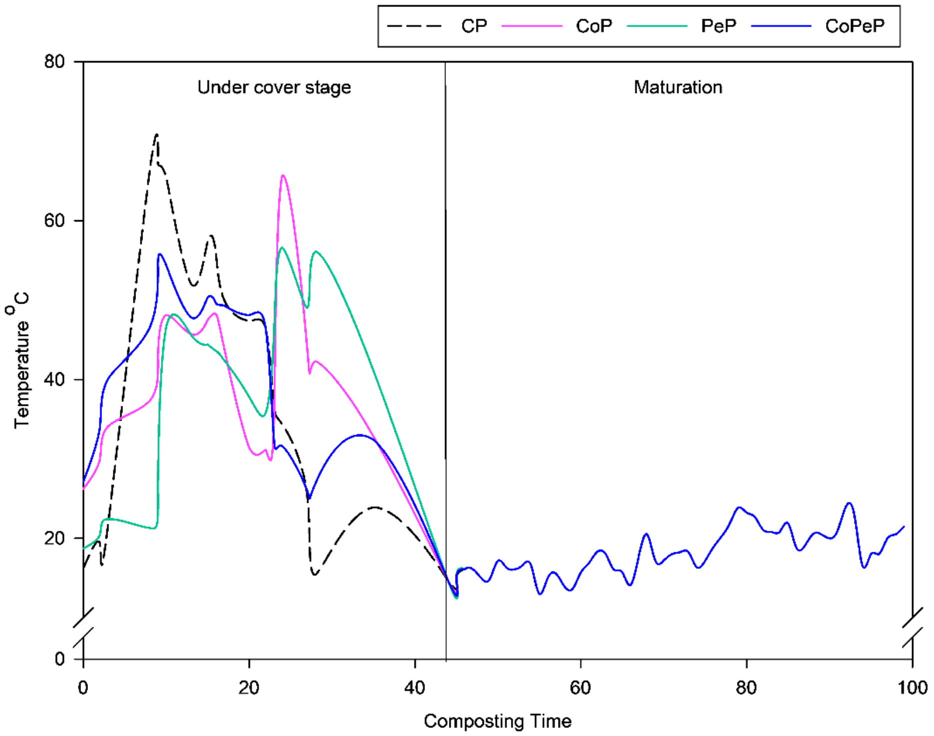

Figure S1. Temperature over composting time at the four treatments: CP: Control Pile, CoP: Covered Pile, PeP: *Penicillium* Pile, and CoPeP: Covered *Penicillium* Pile
